# Safety of Primary Tracheoesophageal Puncture in Patients Submitted to Enlarged Total Laryngectomy with Pectoralis Major Reconstruction

**DOI:** 10.3390/jpm15090435

**Published:** 2025-09-10

**Authors:** Emilia Degni, Sebastiana Lai, Carlo Camillo Ciccarelli, Gamze Yesilli Puzella, Claudia Crescio, Paolo Tropiano, Valeria Fois, Claudio Parrilla, Jacopo Galli, Francesco Bussu

**Affiliations:** 1 Division of Otolaryngology, Azienda Ospedaliera Universitaria, 07100 Sassari, Italy; emilia.degni@aouss.it (E.D.); gamzeyesilli@gmail.com (G.Y.P.); claudia.crescio@aouss.it (C.C.); paolo.tropiano@aouss.it (P.T.); valeria.fois@aouss.it (V.F.); fbussu@uniss.it (F.B.); 2 Department of Medicine, Surgery and Pharmacy, University of Sassari, 07100 Sassari, Italy; carlocamillociccarelli@gmail.com; 3 Speech and Language Therapy Department, School of Health Sciences, Cappadocia University, 50420 Mustafapasa Urgup, Turkey; 4 Division of Otorhinolaryngology, Fondazione Policlinico Universitario A. Gemelli IRCCS, Università Cattolica del Sacro Cuore, 00168 Rome, Italy; claudio.parrilla@policlinicogemelli.it (C.P.); jacopo.galli@policlinicogemelli.it (J.G.); 5 Faculty of Medicine and Surgery, Department of Head and Neck and Sensory Organs, Università Cattolica del Sacro Cuore, Largo F. Vito, 00168 Rome, Italy

**Keywords:** laryngeal squamous cell carcinoma, total laryngectomy, primary tracheoesophageal puncture, voice prosthesis, pharyngocutaneous fistula, dysphagia, contraindications, risk factor, personalized treatment recommendation

## Abstract

**Background/Objectives:** Total laryngectomy (TL) remains a key treatment option for advanced laryngeal cancer. Primary tracheoesophageal puncture (TEP) with voice prosthesis (VP) enables early speech restoration and is increasingly adopted, even in patients with conditions traditionally considered contraindications, such as prior/adjuvant radiotherapy, extended resections, and immediate reconstructive procedures. This study evaluates complication rates and long-term outcomes associated with primary TEP in these settings. **Methods:** A retrospective cohort of 101 patients undergoing TL for laryngeal or hypopharyngeal squamous cell carcinoma at the University Hospital of Sassari (August 2017–December 2024) was analyzed. Demographic, clinical, surgical, and oncological data were collected, with a particular focus on postoperative early complications and late sequelae and oncological outcomes. **Results:** Primary TEP was performed in 78 patients (77.2%). Overall, pharyngocutaneous fistula occurred in 6/101 patients (5.9%), postoperative bleeding in 5/101 (5.0%), and dysphagia in 11/101 (10.9%), with only 2/11 (2.0%) requiring intervention. Mean follow-up was 44.6 ± 3.2 months (median 41, range 4–93). No significant association was found between primary TEP and complication rates, including in patients undergoing enlarged TL with pectoralis major reconstruction. **Conclusions:** Primary TEP appears safe and effective, even in cases requiring extended resections and reconstructive procedures. It should be considered to enhance functional recovery and postoperative quality of life for all motivated patients undergoing total laryngectomy without patient-related contraindications. Our findings may constitute a step towards personalized medicine in laryngeal oncology as they support priortizing patient-specific factors, such as pneumological and neurological clinical conditions and level of cooperation, over purely surgical considerations.

## 1. Introduction

Laryngeal squamous cell carcinoma (LSCC) remains, in many areas, including Italy and Mediterranean Europe, the most common malignancy in the head and neck region [1,2].

The impact on quality of life of surviving patients is all the more significant the more advanced the primary disease is. Traditionally, total laryngectomy has been considered the treatment option with the greatest impact on patients’ quality of life. This is due to the fact that total laryngectomy determines the loss of the sphincteric function of the larynx and consequently of vocal abilities such as speaking, laughing, or crying, as well as the complete separation of the vocal tract, resulting in a permanent tracheostomy and the inability to immerse themselves in water. Additionally, the presence of a tracheostoma in the lower part of the neck obviously has by itself a significant esthetic and therefore social impact on patients who undergo total laryngectomy [3].

Starting from the 1990s, literature evidence has strongly supported, especially in Anglo-Saxon countries, the use of non-surgical organ- and function-preserving approaches that allow for avoiding total laryngectomy even in advanced cases, with the exception of cT4 tumors [4].

However, starting from the 2000s, two main considerations have once again made total laryngectomy increasingly popular, even in primary cases and even when the classification of the primary tumor is below cT4 [3,4,5].

The first consideration is the revaluation of the medium- and long-term functional and oncological outcomes of previous studies that popularized organ and function preservation through non-surgical approaches. In particular, the outcomes of such patients continue to worsen well beyond the follow-up periods of the published studies [6] in terms of survival and, above all, of laryngeal function, which continues to deteriorate for years and even decades after the completion of radiotherapy, despite the anatomical preservation. The concerns around the oncological results of non-surgical organ preservation strategies are supported by the fact that in the cancer statistics of the last decades, laryngeal cancer is the only one, along with adenocarcinoma of the uterine corpus, without a prognostic improvement [7].

On the other hand, the second consideration is that at present, also because of the diffusion of heat and moisture exchange devices and voice prostheses, patients submitted to total laryngectomy can currently achieve a much better quality of life compared to what was possible just a few decades ago, often resulting in voice restoration of excellent quality and a reduction in a whole range of discomforts and issues related to total laryngectomy, including cosmetic and social concerns, since the presence of these heat and moisture exchangers makes the tracheostoma less noticeable and more socially tolerable [8,9].

In particular, voice prostheses (VP) are increasingly being adopted for voice rehabilitation in laryngectomized patients, also due to the progressive reduction in contraindications to tracheoesophageal puncture (TEP). According to the literature, secondary TEP, where the voice prosthesis is placed several months after total laryngectomy (TL), was initially preferred [10,11,12]. However, indications for primary TEP, in which the prosthesis is placed at the time of TL, have steadily increased over time [5,13,14,15].

The aim of the present study is to evaluate the complication rate associated with primary TEP in a cohort of patients who underwent total laryngectomy at a referral center in northern Sardinia, focusing particularly on cases previously considered to have relative or absolute contraindications, such as recurrence after radiotherapy ± chemotherapy and, most notably, extended laryngectomies requiring concomitant reconstructive procedures, in order to better personalize therapeutic recommendations also with attention to quality of life [13].

## 2. Materials and Methods

This retrospective observational cohort study is based on the evaluation of demographic, clinical, oncological, and postoperative data from all patients diagnosed with LSCC, including glottic, supraglottic, and subglottic subsites, and hypopharyngeal squamous cell carcinoma (SCC) who consecutively underwent total laryngectomy at the Otorhinolaryngology Division of the University Hospital of Sassari (AOU Sassari), a tertiary academic referral center in Sardinia, Italy, between August 2017 and December 2024.

### 2.1. Multidisciplinary Standardized Management

At AOU Sassari, all head and neck cancer patients are routinely managed through an integrated clinical pathway, which involves structured discussions by a multidisciplinary team (tumor board) at all stages of care, from initial diagnosis and clinical/radiological staging to treatment planning, toxicity management, and post-treatment follow-up. The tumor board meets weekly and includes specialists in otorhinolaryngology, medical oncology, radiation oncology, radiology, pathology, and, when needed, other healthcare professionals. Each case is jointly evaluated with the goal of a shared definition of staging and the therapeutic path. In the tumor board of the AOU of Sassari, the quality and completeness of the data are ensured by the routine, systematic, and continuous prospective collection of clinical information. All relevant data, from diagnosis to follow-up, are routinely recorded and securely stored in a structured database using REDCap (Research Electronic Data Capture) [16], an open-source platform widely adopted for clinical data management. This approach has provided a reliable and accurate dataset, allowing for methodologically sound retrospective analyses.

All patients who consecutively underwent total laryngectomy (TL) with or without extension to the adjacent structures, including the oropharynx, hypopharynx, thyroid, and skin, were recalled from the above database and included in this study. Demographics, staging, treatment (including surgical details), and follow-up are specifically included in the analysis (see Table 1).

For the purposes of this study, particular attention was given to the correlation between the presence of the voice prosthesis and the occurrence of short-term complications (i.e., postoperative bleeding and pharyngocutaneous fistulae) and long-term sequelae (pharyngoesophageal stricture), as well as to the association between pre-existing conditions that were previously considered contraindications to tracheoesophageal puncture (i.e., salvage surgery after primary surgical/non-surgical treatment, enlarged laryngectomy with or without reconstructive procedures, and adjuvant treatments) and such complications and sequelae.

### 2.2. Technical Notes

The total laryngectomy was performed using a standard technique. In all cases, except for one without neck recurrence, in which the neck dissection had already been performed during a previous partial surgery, the surgery began with a bilateral neck dissection, and it continued with a total laryngectomy, including the thyroid and the level VI lymph nodes in high-risk cases, namely, extralaryngeal spread, subglottic infiltration, and involvement of the pyriform sinus apex and the retrocricoid wall [17]. The thyroid/hemithyroid was removed en-bloc with the larynx when indicated [18].

The opening of the pharynx was always performed at the maximum possible distance from the primary lesion, as previously assessed by endoscopy and imaging; for example in subglottic cases, the larynx was opened at the level of the glossoepiglottic valleculae, preserving the epiglottic mucosa whenever possible.

When possible, the tracheostomy was performed at the end of the dissection of the larynx ± the thyroid from the surrounding structures, particularly from the constrictor muscles (when not involved), to enable a low tracheotomy. Even in cases where a protective tracheotomy was required prior to the total laryngectomy, an effort was still made to perform a low tracheotomy, at least between the second and third rings, but preferably between the third and fourth or the fourth and fifth. This allowed, during the tracheoesophageal puncture phase, for a substantial length of the pharynx and esophagus, at least 2 cm, to be maintained between the pharyngeal opening and the tracheoesophageal puncture, which would thus remain very far away from a potential leakage/fistula from the pharyngeal closure.

In cases with primary closure, a double-layer T-shaped closure of the neopharyngeal opening was performed. The first layer was performed using standard 3-0 Vicryl interrupted sutures, with stitches and knots completely outside the mucosa. The second layer of closure, meant to provide mechanical resistance, was performed using the stumps of pharyngeal constrictor muscle, employing a continuous barbed suture. In cases with extensive skin involvement, extensive involvement of more than 50–60% of the total pharyngeal circumference, or with extensive involvement of the base of the tongue, closure was performed using a myocutaneous or myofascial pectoralis major flap [19].

**Figure 1:**
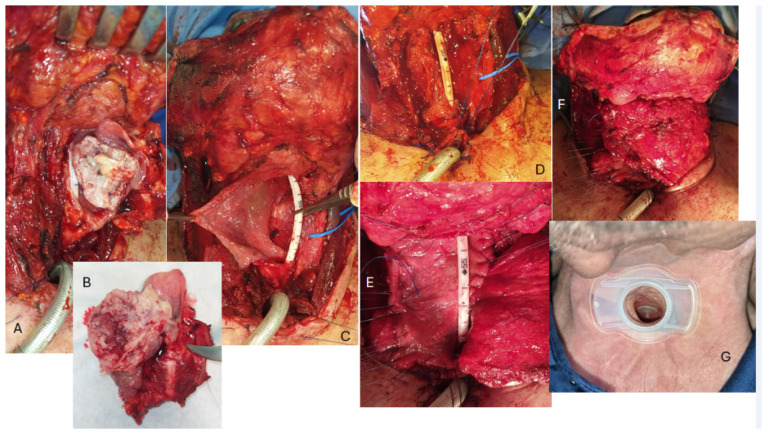
An example of a reconstruction with an inlay insetting of a myofascial pectoralis major flap in a wide pharyngo-laryngeal defect following the removal of a hypopharyngeal carcinoma. (**A**,**B**) Voluminous lesion centred on the left piriform sinus. (**C**) Wide pharyngeal defect; a wide segment of the esophagus (possibly more than 2 cm) is left between the posterior and superior edge of the tracheostoma and the pharyngeal breach. (**D**) The tracheoesophageal puncture is performed with the placement of the voice prosthesis very close to the posterior/superior border of the tracheal mucosa, using the ethilon sutures previously apposed on the cranial/posterior rim of the tracheostoma for traction in order to better stretch and expose the tracheal mucosa itself. (**E**,**F**) The sutures are placed radially and ordered along the edges of the pharyngeal breach, and the pectoralis major is progressively inset to fill the defect and restore the pharyngeal surface. It should be noted that the area of the fascia pectoralis used to fill the pharyngeal defect is significantly larger than what might appear necessary, in consideration of the inevitable scar contraction of the myofascial surface during epithelialization. (**G**) A few months after surgery, the stoma appears stable and the fistula is still comfortably close to the edge.

Atraumatic 3/0 vycril sutures were always placed radially and ordered along the edges of 145 the pharyngeal breach preliminary. The insetting of the pectoralis major flap, brought into the neck through a tunnel between the subcutaneous layer and the clavicle, was always carried out inlay (interpositional technique) [20] (Figure 1).

In all cases in which the patient appeared fit in terms of pneumological, neurological, cognitive, and visual conditions, that is when no patient-related contraindications were present, and had expressed the will and the possibility to manage it with additional accesses to the hospital, a voice prosthesis was primarily placed via tracheoesophageal puncture. Surgery-related contraindications (e.g., previous or possible postoperative irradiation and extended ablative surgery primary reconstruction) never detracted from performing primary TEP and VP placement in the present series.

The TEP was performed closer than what is usually described [21,22], at 0.3 to 0.5 cm from the superior border of posterior tracheal edge. This was performed in order to compensate for the retraction of the posterior edge of the trachea, which usually draws the cutaneous flap inward into the trachea. The main aim of this trick is therefore to facilitate the subsequent replacement of the prosthesis and the management of the fistula itself, which gets to be closer to the stomal opening in the neck surface (see Figure 1D,G).

The pharyngeal myotomy and the section of the external heads of the sternocleidomastoid muscles were never performed primarily.

### 2.3. Follow-Up

Follow-up information was collected from the subsequent clinical visits recorded at our hospital and phone calls in the case of no-shows. For deceased patients, the date and cause of death were recorded, with specific attention to cancer-related mortality. Data concerning tumor recurrence and the development of second primary tumors were also analyzed. Postoperative outcomes were systematically assessed, focusing on early complications, late sequelae, and functional recovery. These included pharyngocutaneous fistula, postoperative bleeding, and dysphagia, particularly if requiring medical or surgical intervention. This comprehensive dataset allowed for an in-depth analysis of both oncological and functional outcomes in the study population.

### 2.4. Statistical Analysis

Preliminary statistical analysis was performed using the JMP software, release 7.0.1, SAS Institute (Cary, NC, USA). To assess the association between the occurrence of complications, such as fistula, postoperative bleeding, or dysphagia, and clinical or surgical variables (e.g., use of a voice prosthesis, prior radiotherapy, and type of reconstruction), contingency and multivariate analyses were performed. A *p*-value < 0.05 was considered statistically significant. All *p*-values were calculated using Pearson’s chi-square and Fisher’s exact tests.

Additional statistical analyses were performed using R (version 4.4.3; R Core Team, 2024) [23] with RStudio software (version 2025.05.1+513; Posit Team, 2023) [24]. Kaplan–Meier survival curves for overall survival (OS), disease-specific survival (DSS), and relapse-free survival (RFS) were generated using the survival and survminer [25] packages. Graphs were combined and annotated with ggplot2 [26], ggpubr [27], dplyr [28], and scales [29]. A post hoc power analysis was performed using the pwr [30] package in R, specifically the function pwr.2p2n.test, o calculate the statistical power for differences between two independent proportions based on Cohen’s h effect size. Multivariable logistic regression models were fitted in R using the broom [31] and dplyr citepref30 packages for model tidying and data manipulation. Odds ratios (ORs) with 95% confidence intervals were derived from model coefficients using the exp() transformation. When no events occurred in one of the exposure groups, estimates were reported as “Not estimable” (NE).

## 3. Results

A total of 101 patients were included in this study. Descriptive statistics of patient and tumor characteristics are shown in Table 1. A total of 10 patients (10%) underwent salvage total laryngectomy (TL) following failure of non-surgical (radiotherapy or chemoradiotherapy) (7 cases) or surgical (3 cases) laryngeal preservation. All the other 91 cases were primary total laryngectomies. Seventy eight out of one hundred and one patients (77.2%) underwent primary TEP and VP placement. No secondary TEP has been recorded in the present series. The most common early post-surgical complication was the development of a pharyngocutaneous fistula, which occurred in six patients (5.9%) and never involved the TEP. Postoperative bleeding was observed in five patients (5.0%). Dysphagia was reported in 11 patients (10.9%) as limitations in the ingestion of food, for example, of certain consistencies, although only 2 patients (2.0%) required specific therapeutic interventions, such as endoscopic dilatation.

**Figure 2:**
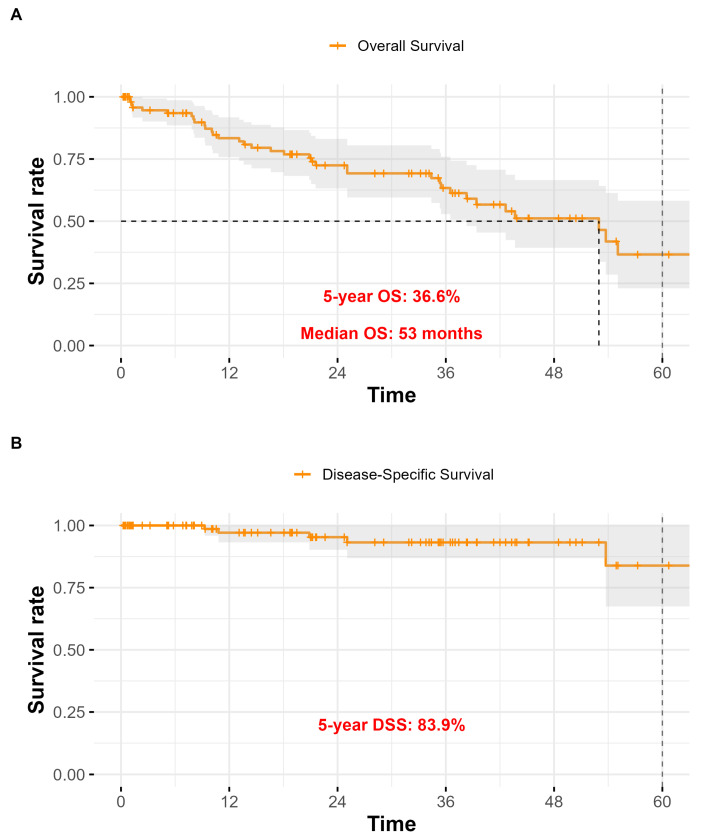

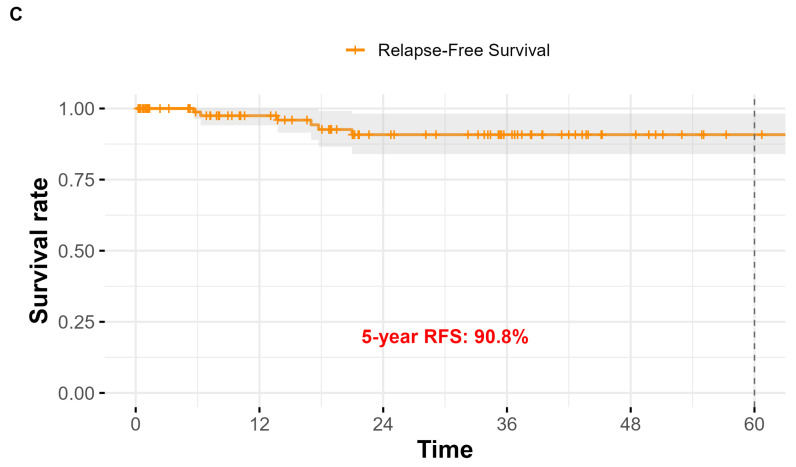
Kaplan–Meier survival curves for the study population (*n* = 101) after total laryngectomy. (**A**) Overall Survival (OS): the estimated 5-year OS rate was 36.6%; (**B**) Disease-Specific Survival (DSS): the 5-year DSS was 83.9%; (**C**) Relapse-Free Survival (RFS): the 5-year RFS was 90.8%.

The overall cumulative rate of relevant early complications and late sequelae was therefore around 10% (Table 1). The mean follow-up duration for the entire cohort was 44.6 ± 3.2 months, with a median of 41 months. Of the 101 patients included in the study, 65 (64.4%) were alive, while 36 (35.6%) had died. At the time of the last observation, the 5-year overall survival (OS) rate was 36.6%. The 5-year disease-specific survival (DSS), considering only deaths attributable to laryngeal carcinoma, was 83.9%. The 5-year relapse-free survival (RFS) was 90.8% (Figure 2).

### 3.1. Postoperative Complications in Relation to Clinical and Surgical Factors

**Figure 3:**
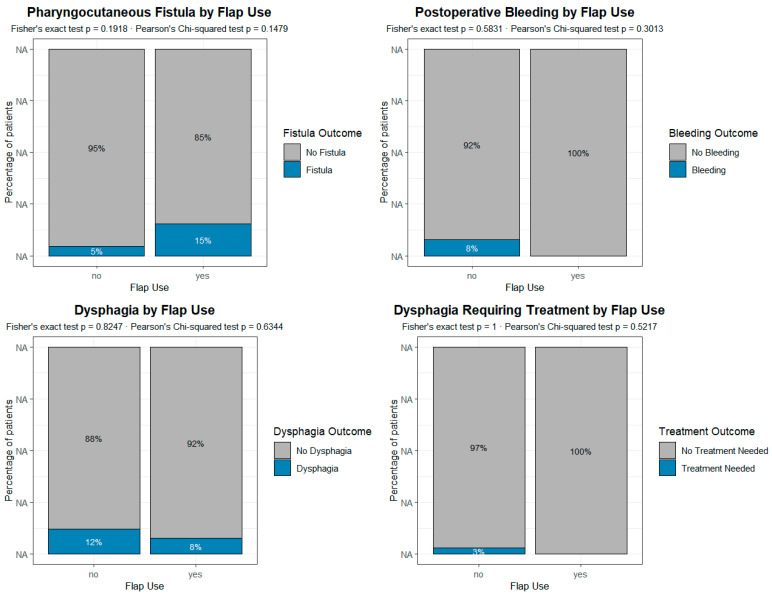
Analysis of the impact of the reconstructive procedure on the different complications and sequelae in patients undergoing primary voice prosthesis placement. According to the present data, the reconstruction with pectoralis major is not associated with any of the most common complications/sequelae. Each plot represents a grouped proportional bar chart, built from contingency tables comparing the incidence of post-surgical complications between patients who underwent flap reconstruction and those who did not. For each complication, the x-axis indicates the presence or absence of flap use (“Flap Use”: Yes/No), while the y-axis represents the percentage of patients within each flap group. Each vertical bar is divided into segments corresponding to the outcomes of the postoperative variable (e.g., “Fistula” vs. “No Fistula”), and the height of each segment reflects its relative proportion within the flap group.

A contingency analysis was preliminarily performed to investigate the potential association between the occurrence of complications/sequelae and several clinical and surgical variables, including prior radio ± chemotherapy, enlargement of laryngectomy, reconstruction with a pectoralis major flap, postoperative radio + chemotherapy, and placement of a voice prosthesis. Overall, the analysis did not reveal any statistically significant associations between the main postoperative complications and the clinical or surgical variables considered (Figure 3). This absence of significant correlations was also confirmed by the multivariate analysis (Table 2).

In particular, according to the present data, the placement of a voice prosthesis through a tracheoesophageal puncture is not a risk factor for the most common complications and sequelae of total laryngectomy.

We therefore analyzed separately the group of 78 patients who underwent primary TEP in the present series in order to assess whether factors that were commonly considered absolute or relative contraindications to such a procedure, namely radiotherapy, extended TL, and reconstructive procedures [32,33,34,35,36], were actually associated with an increased risk of complications and sequelae. We found no such correlation, in particular with pectoralis major reconstruction, as shown in Figure 3.

### 3.2. Power Analysis

A post hoc power analysis was conducted to evaluate the capability of the available sample to detect differences in the incidence of four postoperative complications: pharyngocutaneous fistula, postoperative bleeding, dysphagia, and dysphagia requiring treatment.

Analyses were performed for three predefined clinical comparisons: voice prosthesis (yes vs. no), radiotherapy (yes vs. no), and enlarged resection (yes vs. no). For each outcome and comparison (see Table 3), the number of patients in each group, Cohen’s h effect size, and the corresponding observed power (two-sided, alpha = 0.05) were calculated. The observed power values ranged from 0.05 to 0.58. The highest power was observed for postoperative bleeding in the comparisons involving voice prosthesis and enlarged resection (0.58), while all other analyses showed considerably lower power. These findings indicate that, given the available sample sizes, the study was underpowered to detect small-to-moderate differences in complication rates, and only large differences would likely have been detected.

## 4. Discussion

This study suggests that primary tracheoesophageal puncture (TEP) can be safely performed in all patients undergoing total laryngectomy (TL), including those who have historically been considered unsuitable, or at least high-risk, candidates, such as patients treated with primary or adjuvant radiotherapy and especially those requiring extended laryngectomy with the need for immediate reconstruction. Our results confirm the reliability of the pectoralis major flap in this context, showing no increase in fistula or stenosis rates compared to standard TL without reconstruction, with the potential to improve the personalization of treatment in patients with LSCC.

The most significant finding is therefore that the indication for primary TEP is determined less by surgical factors and more by patient-related aspects such as general health, neurological and cognitive status, motivation, and socioeconomic conditions. This shift toward individualized assessment supports a more personalized approach to decision-making in post-laryngectomy voice rehabilitation. When patients are deemed suitable, the voice prosthesis should be placed primarily, as it offers superior voice quality compared to alternative rehabilitation methods according to most authors [8,9,37].

In our series, disease-specific survival was consistent with the best results reported in the literature, even considering that most patients underwent TL for advanced-stage LSCCs [3,38,39,40,41,42]. The overall complication rate was also comparable to major series [43,44,45]. The pharyngocutaneous/pharyngotracheal fistula rate was particularly low (5.9%), which can be attributed in part to the high proportion of primary TL cases and likely to the standardized pharyngeal closure technique used in all patients: interrupted extra-mucosal sutures for the first layer and a continuous barbed suture for the second layer. Another technical trick that may have contributed to the low fistula rate is the low tracheotomy and the care taken to keep the TEP far away from the pharyngeal breach (see Figure 1).

A certain degree of dysphagia occurred in 10.9% of patients, but persistent, treatment-requiring dysphagia was rare (2%). In cases where the pharyngeal circumference defect exceeded 50%, reconstruction with a pectoralis major flap, preferably using the “inlay” technique, appears to have contributed to the prevention of stenosis [19,38,46]. This supports the view that in centers treating advanced laryngeal and hypopharyngeal cancers, the option of immediate pectoralis major reconstruction should always be available. Taken together, our data suggest that (1) former surgical contraindications to primary TEP, including prior radiotherapy and extended resections with pectoralis major reconstruction, are not supported by current evidence; (2) primary TEP should be offered to all motivated patients without patient-related contraindications; and (3) the selection process for voice rehabilitation should follow a personalized medicine approach, integrating oncological, functional, and quality-of-life considerations, with an increased weight given to patient-related factors, including patient preference and socioeconomic issues. However, it should be acknowledged that this study is a retrospective single-institution case series with relatively small subgroups, such as patients undergoing reconstructive procedures or salvage laryngectomy. Although no increase in complication rates was observed with primary TEP, the limited sample size reduces the statistical power to detect small differences, and the possibility of a type II statistical error, failing to identify an actual association, cannot be excluded, as also demonstrated by the post hoc power analysis. The possibility of safe primary voice prosthesis placement, combined with favorable oncological outcomes and low complication rates, reinforces the role of TL as a cornerstone of laryngeal oncology, against which organ- and function-preserving approaches must continue to be compared [3,47].

## Figures and Tables

**Table 1:** Descriptive statistics.

Descriptive Item	Summary
Demographics	
Age at TL (years), Mean ± SD	67.6 ± 9.6
Age at TL, Median (Range)	67 (47–86)
Sex, n (%)	
Male	87 (86.1%)
Female	14 (13.9%)
Clinical History	
Alcohol Use, n (%)	
Current	53 (52.5%)
Former	15 (14.9%)
Never	23 (22.8%)
Unknown	10 (9.9%)
Smoking Status, n (%)	
Current	46 (45.5%)
Former	46 (45.5%)
Never	3 (3.0%)
Unknown	6 (6.0%)
Primary Total Laryngectomy, n (%)	91 (90.0%)
Salvage Laryngectomy, n (%)	10 (10.0%)
Non-Surgical Organ Preservation	7 (7.0%)
Surgical Organ Preservation	3 (3.0%)
Tumor Characteristics	
Site of Primary Tumor, n (%)	
Glottic Larynx	46 (45.5%)
Supraglottic Larynx	39 (38.6%)
Hypopharynx	12 (11.9%)
Subglottic Larynx	4 (4.0%)
pTN Staging, n (%)	
pT1b	4 (4.0%)
pT2	21 (20.8%)
pT3	35 (34.6%)
pT4a	41 (40.6%)
pN0	59 (58.4%)
pN1	15 (14.9%)
pN2a	1 (1.0%)
pN2b	6 (5.9%)
pN2c	6 (5.9%)
pN3b	14 (13.9%)
Margins, n (%)	
R0	92 (91.1%)
R1	6 (5.9%)
Close	3 (3.0%)
pStage, n (%)	
I	3 (3.0%)
II	14 (14.0%)
III	29 (29.0%)
IVA	40 (39.0%)
IVB	14 (14.0%)
IVC	1 (1.0%)
Surgery	
Enlarged Laryngectomy, n (%)	
Hypopharynx	17 (16.9%)
Base of Tongue	4 (3.9%)
Skin	1 (1.0%)
Trachea	1 (1.0%)
No	78 (77.2%)
Closure, n (%)	
Pectoralis Major	17 (16.8%)
Primary Closure	84 (83.2%)
Pectoralis Flap Variant, n (%)	
Myocutaneous	7 (6.9%)
Myofascial	10 (9.9%)
Voice Prosthesis, n (%)	
Yes	78 (77.2%)
No	23 (22.8%)
Neck Dissection, n (%)	
Yes	100 (99.0%)
No	1 (1.0%)
Adjuvant treatment, n (%)	
Radiochemotherapy	40 (39.6%)
Radiotherapy	15 (14.9%)
No Adjuvant	46 (45.5%)
Postoperative Complications and Sequelae	
Pharyngocutaneous Fistula, n (%)	
Yes	6 (6.0%)
No	95 (94.0%)
Postoperative Bleeding, n (%)	
Yes	5 (5.0%)
No	96 (95.0%)
Dysphagia, n (%)	
Yes	11 (10.9%)
No	90 (89.1%)
Dysphagia Needing Treatment, n (%)	
Yes	2 (2.0%)
No	99 (98.0%)
Outcomes	
Follow-up (months)	Mean ± SD: 44.6 ± 3.2; Median: 41 (4–93)
Overall Survival (5-year OS)	36.6%
Alive	65 (64.4%)
Deceased	36 (35.6%)
Disease-Specific Survival (5-year DSS)	83.9%
Relapse-Free Survival (5-year RFS)	90.8%
Relapse, n (%)	
Yes	6 (6.0%)
No	95 (94.0%)
Pattern of Relapse, n (%)	
Locoregional	2 (2.0%)
Locoregional and Distant	1 (1.0%)
Regional	3 (3.0%)
No Relapse	95 (94.0%)
Second Primary Tumor (SPT), n (%)	
Yes	19 (18.8%)
No	82 (81.2%)

Descriptive statistics including demographic data, clinical history, tumor characteristics, surgical details, postoperativecomplications of the study population, and oncologic outcomes.

**Table 2:** Multivariable logistic regression analysis of postoperative complications.

Complication	Variable	OR	95% CI	*p*-Value
Pharyngocutaneous fistula	Voice prosthesis (yes vs. no)	1.82	0.26–37.8	0.606
	Radiotherapy/radiochemotherapy	0.31	0.029–2.23	0.261
	Enlarged resection	NE	NE	0.995
	Flap reconstruction	NE	NE	0.995
Postoperative bleeding	Voice prosthesis (yes vs. no)	NE	NE	0.996
	Radiotherapy/radiochemotherapy	0.48	0.059–3.11	0.440
	Enlarged resection	NE	NE	0.998
	Flap reconstruction	NE	NE	1.000
Dysphagia	Voice prosthesis (yes vs. no)	1.37	0.32–9.51	0.701
	Radiotherapy/radiochemotherapy	0.80	0.18–3.54	0.756
	Enlarged resection	1.91	0.089–16.3	0.594
	Flap reconstruction	0.67	0.052–16.3	0.765
Dysphagia needing treatment	Voice prosthesis (yes vs. no)	NE	NE	0.997
	Radiotherapy/radiochemotherapy	NE	NE	0.997
	Enlarged resection	8.25	0.289–239	0.162
	Flap reconstruction	NE	NE	0.998

The table shows odds ratios (ORs) with 95% confidence intervals (CIs) and *p*-values for the association between voice prosthesis use, prior radiotherapy/radiochemotherapy, enlarged resection, and reconstructive flap with four postoperative complications: pharyngocutaneous fistula, postoperative bleeding, dysphagia, and dysphagia requiring treatment. None of the examined variables showed a statistically significant association with any complication (all *p*-values > 0.05). In some cases, ORs could not be estimated (NE) due to the absence of events in one of the comparison groups, resulting in wide or undefined confidence intervals.

**Table 3:** Post hoc power analysis for the association between surgical/treatment variables and postoperative complications.

Outcome	Comparison Group	n1	n2	Cohen’s *h*	Power
Pharyngocutaneous fistula	Voice prosthesis (yes vs. no)	23	78	0.0918	0.067
Pharyngocutaneous fistula	Radiotherapy (yes vs. no)	35	66	0.1645	0.123
Pharyngocutaneous fistula	Enlarged resection (yes vs. no)	78	23	0.1418	0.092
Postoperative bleeding	Voice prosthesis (yes vs. no)	23	78	0.5119	0.578
Postoperative bleeding	Radiotherapy (yes vs. no)	35	66	0.2443	0.215
Postoperative bleeding	Enlarged resection (yes vs. no)	78	23	0.5119	0.578
Dysphagia	Voice prosthesis (yes vs. no)	23	78	0.0945	0.068
Dysphagia	Radiotherapy (yes vs. no)	35	66	0.0263	0.052
Dysphagia	Enlarged resection (yes vs. no)	78	23	0.0870	0.066
Dysphagia requiring treatment	Voice prosthesis (yes vs. no)	23	78	0.3216	0.273
Dysphagia requiring treatment	Radiotherapy (yes vs. no)	35	66	0.3499	0.387
Dysphagia requiring treatment	Enlarged resection (yes vs. no)	78	23	0.1932	0.129

Observed power (two-sided, α = 0.05) computed with pwr.2p2n.test in R, based on Cohen’s *h* and actual group sizes for each comparison (yes vs. no).

## Data Availability

The data presented in this study are available on request from the corresponding author.

## Abbreviations

The following abbreviations are used in this manuscript:

LSCC	Laryngeal Squamous Cell Carcinoma
SCC	Squamous Cell Carcinoma
TL	Total Larygectomy
HME	Heat and Moisture Exchange
VP	Voice Prosthesis
TEP	Tracheoesophageal Puncture
OS	Overall Survival
DSS	Disease-Specific Survival
RFS	Relapse-Free Survival
IRB	Institutional Review Board
REDCap	Research Electronic Data Capture
SPT	Second Primary Tumor
VIF	Variance Inflation Factor
CIs	Confidence Intervals
NE	Not Estimable
