# Correction: Degni et al. Safety of Primary Tracheoesophageal Puncture in Patients Submitted to Enlarged Total Laryngectomy with Pectoralis Major Reconstruction. *J. Pers. Med.* 2025, *15*, 435

**DOI:** 10.3390/jpm15120627

**Published:** 2025-12-15

**Authors:** Emilia Degni, Sebastiana Lai, Carlo Camillo Ciccarelli, Gamze Yesilli Puzella, Claudia Crescio, Paolo Tropiano, Valeria Fois, Claudio Parrilla, Jacopo Galli, Francesco Bussu

**Affiliations:** 1 Division of Otolaryngology, Azienda Ospedaliera Universitaria, 07100 Sassari, Italy; emilia.degni@aouss.it (E.D.); gamzeyesilli@gmail.com (G.Y.P.); claudia.crescio@aouss.it (C.C.); paolo.tropiano@aouss.it (P.T.); valeria.fois@aouss.it (V.F.); fbussu@uniss.it (F.B.); 2 Department of Medicine, Surgery and Pharmacy, University of Sassari, 07100 Sassari, Italy; carlocamillociccarelli@gmail.com; 3 Speech and Language Therapy Department, School of Health Sciences, Cappadocia University, 50420 Mustafapasa Urgup, Turkey; 4 Division of Otorhinolaryngology, Fondazione Policlinico Universitario A. Gemelli IRCCS, Università Cattolica del Sacro Cuore, 00168 Rome, Italy; claudio.parrilla@policlinicogemelli.it (C.P.); jacopo.galli@policlinicogemelli.it (J.G.); 5 Faculty of Medicine and Surgery, Department of Head and Neck and Sensory Organs, Università Cattolica del Sacro Cuore, Largo F. Vito, 00168 Rome, Italy

## Figure Legend

In the original publication [1], there was a mistake in the legend for Figure 3. Specifically, the color coding in the bar plots was inverted: “Fistula” was displayed in grey instead of blue, and “No fistula” was displayed in blue instead of grey. The same inversion occurred for the remaining three postoperative complications represented in Figure 3.

The correct Figure 3 legend appears below:

**Figure 3:**
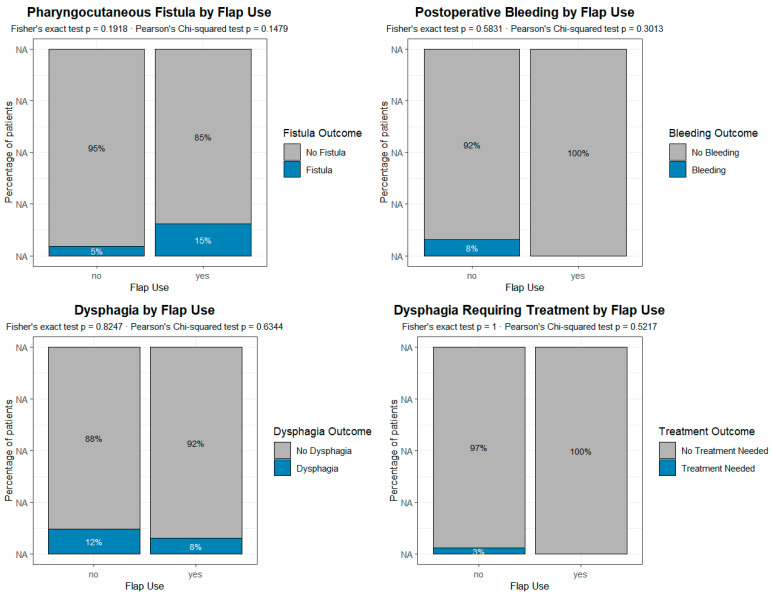
Analysis of the impact of the reconstructive procedure on the different complications and sequelae in patients undergoing primary voice prosthesis placement. According to the present data, the reconstruction with pectoralis major is not associated with any of the most common complications/sequelae. Each plot represents a grouped proportional bar chart, built from contingency tables comparing the incidence of post-surgical complications between patients who underwent flap reconstruction and those who did not. For each complication, the x-axis indicates the presence or absence of flap use (“Flap Use”: Yes/No), while the y-axis represents the percentage of patients within each flap group. Each vertical bar is divided into segments corresponding to the outcomes of the postoperative variable (e.g., “Fistula” vs. “No Fistula”), and the height of each segment reflects its relative proportion within the flap group.

## Error in Table

In the original publication [1], there were some mistakes in Table 1 as published.

- **Pharyngocutaneous Fistula, n (%):** The percentage reported for “Yes” has been corrected from “3.0%” to “6.0%”; (%): the percentage reported for “No” has been corrected from “94.1%” to “94.0%”.
- **Relapse, n (%):** The percentage reported for relapse cases was based on a preliminary version of the dataset and did not reflect the final validated data used for the statistical analyses. This discrepancy was also evident in the subsequent section, “Pattern of Relapse, n (%)”, where the total number of relapse cases did not match the value shown in the table. Specifically, the number and percentage of “Yes” has been corrected from “11 (11.1%)” to “6 (6.0%)”; the number and percentage of “No” has been corrected from “88 (88.9%)” to “95 (94.0%)”.
- **pStage, n (%):** The percentages reported for stages II, III, IVA, and IVB were incorrectly calculated in the published version. Specifically, the percentage of stage II has been corrected from “13.87%” to “14.0%”; the percentage of stage III has been corrected from “28.7%” to “29.0%”; the percentage of stage IVA has been corrected from “39.6%” to “39.0%”; the percentage of stage IVB has been corrected from “13.9%” to “14.0%”.

The corrected Table 1 appears below:

**Table 1:** Descriptive statistics.

Descriptive Item	Summary
Demographics	
Age at TL (years), Mean ± SD	67.6 ± 9.6
Age at TL, Median (Range)	67 (47–86)
Sex, n (%)	
Male	87 (86.1%)
Female	14 (13.9%)
Clinical History	
Alcohol Use, n (%)	
Current	53 (52.5%)
Former	15 (14.9%)
Never	23 (22.8%)
Unknown	10 (9.9%)
Smoking Status, n (%)	
Current	46 (45.5%)
Former	46 (45.5%)
Never	3 (3.0%)
Unknown	6 (6.0%)
Primary Total Laryngectomy, n (%)	91 (90.0%)
Salvage Laryngectomy, n (%)	10 (10.0%)
Non-Surgical Organ Preservation	7 (7.0%)
Surgical Organ Preservation	3 (3.0%)
Tumor Characteristics	
Site of Primary Tumor, n (%)	
Glottic Larynx	46 (45.5%)
Supraglottic Larynx	39 (38.6%)
Hypopharynx	12 (11.9%)
Subglottic Larynx	4 (4.0%)
pTN Staging, n (%)	
pT1b	4 (4.0%)
pT2	21 (20.8%)
pT3	35 (34.6%)
pT4a	41 (40.6%)
pN0	59 (58.4%)
pN1	15 (14.9%)
pN2a	1 (1.0%)
pN2b	6 (5.9%)
pN2c	6 (5.9%)
pN3b	14 (13.9%)
Margins, n (%)	
R0	92 (91.1%)
R1	6 (5.9%)
Close	3 (3.0%)
pStage, n (%)	
I	3 (3.0%)
II	14 (14.0%)
III	29 (29.0%)
IVA	40 (39.0%)
IVB	14 (14.0%)
IVC	1 (1.0%)
Surgery	
Enlarged Laryngectomy, n (%)	
Hypopharynx	17 (16.9%)
Base of Tongue	4 (3.9%)
Skin	1 (1.0%)
Trachea	1 (1.0%)
No	78 (77.2%)
Closure, n (%)	
Pectoralis Major	17 (16.8%)
Primary Closure	84 (83.2%)
Pectoralis Flap Variant, n (%)	
Myocutaneous	7 (6.9%)
Myofascial	10 (9.9%)
Voice Prosthesis, n (%)	
Yes	78 (77.2%)
No	23 (22.8%)
Neck Dissection, n (%)	
Yes	100 (99.0%)
No	1 (1.0%)
Adjuvant treatment, n (%)	
Radiochemotherapy	40 (39.6%)
Radiotherapy	15 (14.9%)
No Adjuvant	46 (45.5%)
Postoperative Complications and Sequelae	
Pharyngocutaneous Fistula, n (%)	
Yes	6 (6.0%)
No	95 (94.0%)
Postoperative Bleeding, n (%)	
Yes	5 (5.0%)
No	96 (95.0%)
Dysphagia, n (%)	
Yes	11 (10.9%)
No	90 (89.1%)
Dysphagia Needing Treatment, n (%)	
Yes	2 (2.0%)
No	99 (98.0%)
Outcomes	
Follow-up (months)	Mean ± SD: 44.6 ± 3.2; Median: 41 (4–93)
Overall Survival (5-year OS)	36.6%
Alive	65 (64.4%)
Deceased	36 (35.6%)
Disease-Specific Survival (5-year DSS)	83.9%
Relapse-Free Survival (5-year RFS)	90.8%
Relapse, n (%)	
Yes	6 (6.0%)
No	95 (94.0%)
Pattern of Relapse, n (%)	
Locoregional	2 (2.0%)
Locoregional and Distant	1 (1.0%)
Regional	3 (3.0%)
No Relapse	95 (94.0%)
Second Primary Tumor (SPT), n (%)	
Yes	19 (18.8%)
No	82 (81.2%)

Descriptive statistics including demographic data, clinical history, tumor characteristics, surgical details, postoperative complications of the study population, and oncologic outcomes.

The authors state that the scientific conclusions are unaffected. This correction was approved by the Academic Editor. The original publication has also been updated.
